# Quercetin Inhibits Hsp70 Blocking of Bovine Viral Diarrhea Virus Infection and Replication in the Early Stage of Virus Infection

**DOI:** 10.3390/v14112365

**Published:** 2022-10-26

**Authors:** Nannan Chen, Yu Liu, Tongtong Bai, Jinwei Chen, Zhibo Zhao, Jing Li, Baihui Shao, Zecai Zhang, Yulong Zhou, Xue Wang, Zhanbo Zhu

**Affiliations:** 1 College of Animal Science and Veterinary Medicine, Heilongjiang Bayi Agricultural University, Daqing 163319, China; 2 Key Laboratory of Bovine Disease Control in Northeast China, Ministry of Agriculture and Rural Affairs, Daqing 163319, China; 3 Branch of Animal Husbandry and Veterinary of Heilongjiang Academy of Agricultural Sciences, Qiqihar 161006, China; 4 Heilongjiang Provincial Engineering Research Center for Prevention and Control of Cattle Diseases, Heilongjiang Bayi Agricultural University, Daqing 163319, China

**Keywords:** BVDV, flaviviruses, antiviral, quercetin, Hsp70, inhibitor, oxidative stress

## Abstract

Bovine viral diarrhea virus (BVDV), a positive-strand RNA virus of the genus Pestivirus in the Flaviviridae family, is the causative agent of viral diarrheal disease in bovine. BVDV has been used as a surrogate model for the hepatitis C virus (HCV) to evaluate the efficacy of antiviral drugs. The plant flavonol quercetin causes multiple health-promoting effects in humans and animals. It can be made into a variety of additives, and it exerts a variety of immunomodulatory effects with the potential to be used as an antiviral agent. However, quercetin’s antiviral effect and mechanism of action on BVDV are still unclear. Therefore, this study was designed to evaluate quercetin’s effect on BVDV virus replication in vitro and in vivo and elucidate its mechanism of action. A CCK-8 kit was used to analyze the toxicity of the quercetin to the MDBK cells. Western blot, qRT-PCR, TCID_50_, and histological analysis were used to determine the mechanism of quercetin’s anti-BVDV activity. An oxidative stress kit was used to evaluate the effects of quercetin on ROS, antioxidant enzymes, and MDA indexes. The effect of quercetin on IL-2 and IFN-γ in the serum of mice was determined by using an ELISA kit. The results showed that quercetin inhibits Hsp70, blocks BVDV infection in the early stage of virus infection and inhibits BVDV replication by inhibiting oxidative stress or ERK phosphorylation. In addition, quercetin alleviated the decrease in IFN-γ and IL-2 in the serum of BVDV-infected mice. Quercetin ameliorated BVDV-induced histopathological changes. In summary, this study demonstrated for the first time the role of Hsp70 in BVDV infection and the potential application of quercetin in treating BVDV infection.

## 1. Introduction

Bovine viral diarrhea (BVD) is widely distributed worldwide, especially in countries with developed cattle industries. It is one of the important diseases that cause economic losses in the cattle industry with a high seropositive rate. Bovine viral diarrhea virus (BVDV), a positive-strand RNA virus of the genus Pestivirus in the Flaviviridae family, is the causative agent of viral diarrheal disease in bovines. Flaviviridae also includes critical human pathogens, such as West Nile virus (WNV), hepatitis C virus (HCV), yellow fever virus (YFV), dengue virus (DV), and Saint Louis encephalitis virus (SLEV) [1]. Acute infection of BVDV is associated with immune dysfunction and can cause peripheral blood lymphopenia and apoptosis. It has two biotypes, cytopathic (cp) and non-cytopathic (ncp), based on the effect of BVDV infection on cells [2]. BVDV is one of the cattle’s most economically important viruses, but this pathogen can also infect pigs and a wide range of domestic and wild ruminants [3]. According to the literature, BVDV has been used as a surrogate model for the hepatitis C virus (HCV) to evaluate the efficacy of antiviral drugs [4]. Treatment with antiviral drugs is an attractive strategy to prevent infection in newborn calves [5]. However, few antiviral drugs are currently available for treating BVDV (cp/ncp) infection [6].

Quercetin is one of the most prominent dietary flavonoids, which is ubiquitously present in foods and vegetables [7]. Quercetin can be made into various additives because of its stable chemical structure and water-soluble derivatives (including feed additives for livestock and poultry) [8]. Multiple studies have shown that quercetin has a strong anti-inflammatory, antioxidant, immunomodulatory, and antiviral effect [9,10,11]. It inhibits viral infection at multiple stages, including endocytosis, transcription of the viral genome, and synthesis of viral proteins. Recent studies have shown that quercetin is the first compound that specifically inhibits EBOV VP24 IFN-1-inhibitory function, restoring the IFN signaling cascade, and leading to the block of viral infection [12]. Quercetin exhibits antiviral activity against several zoonotic coronaviruses, including SARS-CoV-2, and can be used as a potential compound for developing anti-coronavirus drugs [13]. In addition, quercetin has been shown to have anti-hepatitis properties for the hepatitis B virus (HBV) [14]. Therefore, quercetin is a promising candidate for developing antiviral drugs against multiple viruses.

Quercetin also affects the expression of heat shock protein 70 (Hsp70) [15]. As one of the most abundant HSPs, Hsp70 functions as an intracellular chaperone/modulator of apoptosis and a stimulator of adaptive and innate immunity. In addition, inhibition of Hsp70 can also enhance the induction function of IFN [16]. Thus, Hsp70 is considered a logical target for anticancer therapy and the prevention of immune escape [17]. Studies have shown that members of the Flaviviridae family, including swine fever, ZIKA, and dengue viruses, can interact with Hsp70 [18,19,20]. More importantly, studies have confirmed that enhanced ERK phosphorylation and products of oxidative stresses, e.g., ROS, are increased during infection with BVDV [21,22]. There is a positive correlation between the phosphorylation of Hsp70 and the activity of ERK, and Hsp70 regulates oxidative stress-mediated cell damage and death, while quercetin has antiviral and antioxidant effects [23,24]. At the same time, the molecular target of quercetin is ERK [11]. Thus, we evaluated the effect of quercetin on BVDV virus replication in vitro and in vivo to identify the potential antiviral targets of quercetin and its mechanism of action. This study provides a fundamental basis for exploring the molecular mechanism of immunosuppression by natural antiviral drugs during BVDV infection.

## 2. Materials and Methods

### 2.1. Animals and Reagents

BALB/c mice were purchased from Beijing Weitong Lihua Laboratory Animal Technology Co., Ltd. (Beijing, China). (6–8 weeks old, 18–22 g). The animal study was reviewed and approved by the Management Committee of the Experimental Animal Center of Heilongjiang Bayi Agricultural University (DWKJXY2022029). The cp BVDV-1a (strain NADL, No. VR-534) and ncp BVDV-1b (strain NY-1, No. VR-524) were derived from the American Type Culture Collection (ATCC, Manassas, VA, USA). Reactive oxygen species (ROS), total antioxidant capacity (T-AOC), superoxide dismutase (SOD), catalase (CAT), glutathione peroxidase (GSH-Px), and malondialdehyde (MDA) were measured by using commercial kits (Nanjing Jiancheng Bioengineering Institute, Nanjing, China). Cell Counting Kit 8 (WST-8/CCK8) was purchased from Abcam (ab228554). Quercetin was purchased from Chengdu Must Bio-Technology Co., Ltd. and was resuspended in DMSO. Hsp70 lentivirus was purchased from Hanbio Technology Co., Ltd. The production of IL-2 and IFN-γ were measured by using commercial ELISA kits (IL-2, SEA073Mu, USCN Life Science, Wuhan, China; IFN-γ, SEA049Mu, USCN Life Science, Wuhan, China).

### 2.2. Toxicity of Quercetin on MDBK Cells

The concentration of stock quercetin solution was 100 mmol/L. MDBK cells at a density of 1 × 10^4^ were seeded on 96-well plates, and stock quercetin solution was added at a final concentration of 20, 25, 50, and 100 μmol/L, respectively. DMSO was used as a control. After 48 h of culture, a CCK-8 kit was used to detect the toxicity of the drug to the cells in each well using a microplate reader according to the manufacturer’s instructions.

### 2.3. Measurement of Quercetin’s Antiviral Activity

To evaluate the inhibitory effect of quercetin on the virus, the virus titer in MDBK cells was measured by TCID_50_. First, the same volume of quercetin (20, 25, 50, 100 μmol/L) and (cp/ncp) BVDV virus were added to MDBK cells, and then the supernatant of the infected cells was collected. After tenfold serial dilution, the supernatant was inoculated onto MDBK cells, and the cells were grown to 80% confluence. Forty-eight hours after infection, cytopathic lesions were scored and TCID_50_ values were calculated using the Reed–Muench method [25].

### 2.4. Determining the Effects of Quercetin on Hsp70 Expression and BVDV Replication

In the first group of experiments, MDBK cells were treated with different concentrations of quercetin (20, 25, 50, 100 μmol/L) for 2 h. Subsequently, BVDV (cp/ncp) stock solution was added to reach an MOI of 1 and incubated for 1 h for adsorption. Finally, DMEM complete medium was added. In the second set of experiments, the virus was adsorbed and incubated with quercetin and a maintenance medium. After 48 h of infection, viral RNA, protein expression, and BVDV yields were analyzed by RT-qPCR, Western blot, and viral titers.

### 2.5. Determining the Effects of Hsp70 Overexpression on BVDV Replication

MDBK cells at a density of 3 × 10^5^/mL were prepared, and when the cell confluence reached 30–50%, the lentivirus overexpressing Hsp70 was used to infect the cells according to the manufacturer’s instructions. Fluorescence was observed every 24 h, and after 48 h, the cells were infected with BVDV (cp/ncp). The effects of Hsp70 on viral replication were determined at 12, 24, and 48 h after infection.

### 2.6. Determining the Effects of Hsp70 SiRNA on BVDV Replication

MDBK cells prepared similarly were transfected with Hsp70 siRNA (Hanbio Technology Co., Ltd., Harbin, China) according to the manufacturer’s instructions. After 24 h of transfection, the cells were infected with BVDV (cp/ncp), and the inhibitory effect of Hsp70 siRNA on the viral replication was determined after 48 h.

### 2.7. Determining the Effects of Quercetin and Hsp70 SiRNA on the Expression of Antioxidant Genes in MDBK Cells

MDBK cells were treated with 100 μmol/L quercetin or Hsp70 siRNA for 24 h and were then infected with BVDV (cp/ncp) with DMSO being used as a control group. RNA was extracted after 48 h, and the expressions of antioxidant-related genes NQO1, HO-1, and Nrf2 were determined by RT-PCR (Table 1).

### 2.8. Determining the Effects of Quercetin and Hsp70 SiRNA on Intracellular ROS in MDBK Cells

MDBK cells were treated with 100 μmol/L quercetin or Hsp70 siRNA for 24 h and were then infected with BVDV (cp/ncp). After washing, DMEM complete medium was added to the culture for 24 h. The cells were washed, and DCFH-DA was added and incubated in the dark for 15 min. The cells were washed three times with serum-free DMEM medium, and the cells were collected to prepare a single-cell suspension. OD value was measured by a fluorescence spectrophotometer at a wavelength of 550 nm.

### 2.9. Determining the Effects of Quercetin and Hsp70 SiRNA on Antioxidant Enzymes and MDA Indexes in MDBK Cells

MDBK cells were treated with 100 μmol/L quercetin or Hsp70 siRNA for 24 h. After washing twice, the cells were disrupted on ice with an ultrasonic cell crusher. Subsequently, the concentrations of T-AOC, SOD, CAT, GSH-Px, and MDA were measured by commercial kits according to the manufacturer’s instructions [26].

### 2.10. Determining the Effects of Quercetin and Hsp70 SiRNA on the ERK Pathway

MDBK cells were seeded in 12-well plates at a density of approximately 3 × 10^5^/mL. When the cells reached a confluence of 30–50%, 100 μmol/L of quercetin or Hsp70 siRNA were added and incubated for 24 h. QCells were then infected with BVDV (cp/ncp) for 48 h, and protein samples were collected to determine the effects of quercetin and Hsp70 on the expression levels of ERK/PERK in MDBK cells.

### 2.11. BVDV Animal Infection Model

In our previous studies, we established a mouse model of BVDV infection [1]. In this study, we divided the mice into cp BVDV infection group, ncp BVDV infection group, control group, 50 mg/kg quercetin administration group, 100 mg/kg quercetin administration group, DMSO control group, 50 mg/kg quercetin + cp BVDV group, 50 mg/kg quercetin + ncp BVDV group, 100 mg/kg quercetin + cp BVDV group, and 100 mg/kg quercetin + ncp BVDV group. Each group had six mice.

The virus infection group received an intraperitoneal injection of a total dose of 0.4 mL (10^6^ copies/mL) of the virus. Control mice were treated with 0.4 mL DMEM by IP injection (Gibco, Grand Island, NY, USA). Quercetin was administered by gavage at 0.2 mL/d for 7 consecutive days. On the 7th day after virus infection, blood samples were collected from the eyeball, and then all mice were sacrificed by cervical dislocation.

### 2.12. In Vivo Virus Replication Assay

In order to assess the viral replication in mouse blood, the nucleotide sequence of the BVDV 5′ non-coding region (NCR) was amplified and a template standard of known copy number was used as described previously [1]. SYBR Premixed ExTaqII (RR820A, TaKaRa Biotechnology, Dalian, China) was used for PCR following the manufacturer’s instructions. Quantitative real-time PCR (qRT-PCR) (Bio-Rad, Hercules, CA, USA) was used to determine the copy number of the BVDV 5′NCR gene. Results were shown as mean copies per milliliter of the whole blood.

### 2.13. Analysis of the IL-2 and IFN-γ Concentration In Vivo

In order to examine the effect of quercetin on IL-2 and IFN-γ in the blood of BVDV-infected mice, blood samples were collected from the eyeballs of mice on the 7th day of infection, and the serum was obtained by centrifugation. The concentrations of IL-2 and IFN-γ in serum were measured using mouse IL-2 (SEA073Mu, USCN Life Sciences, Wuhan, China) and IFN-γ (SEA049Mu, USCN Life Sciences, Wuhan, China) ELISA kits according to the manufacturer’s instructions. All samples were measured in triplicates.

### 2.14. Analysis of Antioxidant Gene Expression In Vivo

According to the method described above, the effect of quercetin treatment on antioxidant gene expression in BVDV-infected mice was analyzed by qRT-PCR. Blood samples were collected from mouse eyeballs, RNA was extracted, and cDNA was reverse transcribed. Each sample was measured in triplicates.

### 2.15. Analysis of Oxidative Stress and Antioxidant Enzymes In Vivo

The effects of quercetin on oxidative stress (ROS), malondialdehyde (MDA), and antioxidant enzymes T-AOC, T-SOD, CAT, and GSH-Px in BVDV-infected mice were analyzed by using commercial kits.

### 2.16. Histological Analysis

Portions of the duodenum and spleen of mice in all experimental groups were fixed in 4% paraformaldehyde. The tissue sections were prepared by washing, dehydration, transparency, waxing, embedding, sectioning, spreading, hematoxylin–eosin (H&E) staining, and sealing. The histopathological changes in the duodenum and spleen were examined with a microscope (Ezhou, China).

### 2.17. Statistical Analysis

Statistical analysis was performed using GraphPad Prism version 8.0 (GraphPad software). All data were expressed as mean ± SD, *p* < 0.05 indicated statistically significant difference. All samples were analyzed in triplicate.

## 3. Results

### 3.1. Quercetin Inhibits Hsp70 Expression and BVDV Replication

**Figure 1:**
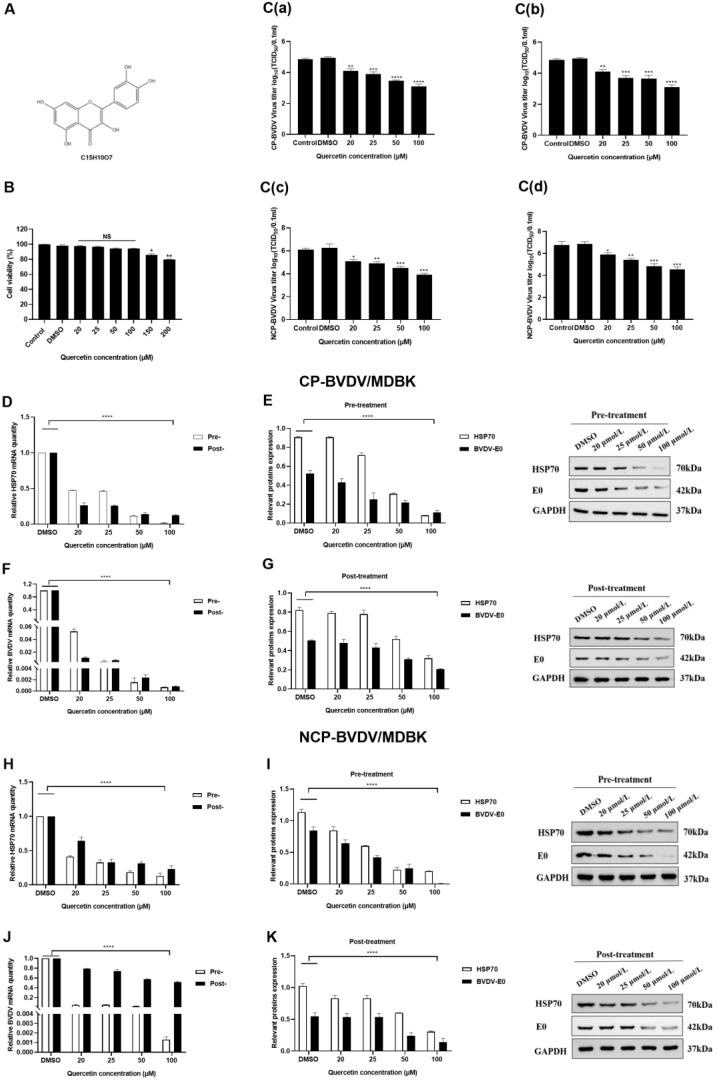
Quercetin inhibits Hsp70 expression and BVDV replication. (**A**): Chemical structure of quercetin is shown. (**B**): Quercetin on MDBK cell viability. (**C**): TCID_50_ of quercetin against the cp-BVDV virus ((**a**): before infection; (**b**): after infection). (**C**): TCID_50_ of quercetin against the ncp-BVDV virus ((**c**): before infection; (**d**): after infection). (**D**–**G**): Inhibitory effect of quercetin on Hsp70 and BVDV in cp-BVDV-infected MDBK cells. (**H**–**K**): Inhibitory effect of quercetin on Hsp70 and BVDV in ncp-BVDV-infected MDBK cells. Pre: pre-treatment; Post: Post-treatment. **** *p <* 0.0001, *** *p <* 0.001, ** *p <* 0.01, * *p <* 0.05, *n* = 3. Data are presented as mean ± SD.

In the initial experiment, we determined the safety range of quercetin concentrations by using the CCK8 method. Minimum cytotoxicity was observed when the concentration of quercetin ranging from 20 to 100 μmol/L was used (Figure 1B). Thus, this concentration range was used for subsequent studies. To determine whether quercetin functions in the entry or post-entry phase of BVDV infection, quercetin was added separately before and after viral infection. Dose-dependent reductions in viral titers were observed in both experiments (Figure 1C). Expression of Hsp70 and BVDV mRNA in cells with the addition of quercetin before infection was significantly lower than that in cells with the addition of quercetin after infection. The most significant inhibitory effect was observed when the concentration of quercetin reached 100 μmol/L. Furthermore, the inhibitory effect of quercetin on cp-BVDV was significantly higher than that of ncp-BVDV (Figure 1D–K). These results suggest that quercetin and Hsp70 may play a role at both the entry and post-entry levels of BVDV infection.

### 3.2. Hsp70 Positively Regulates the Production of BVDV

**Figure 2:**
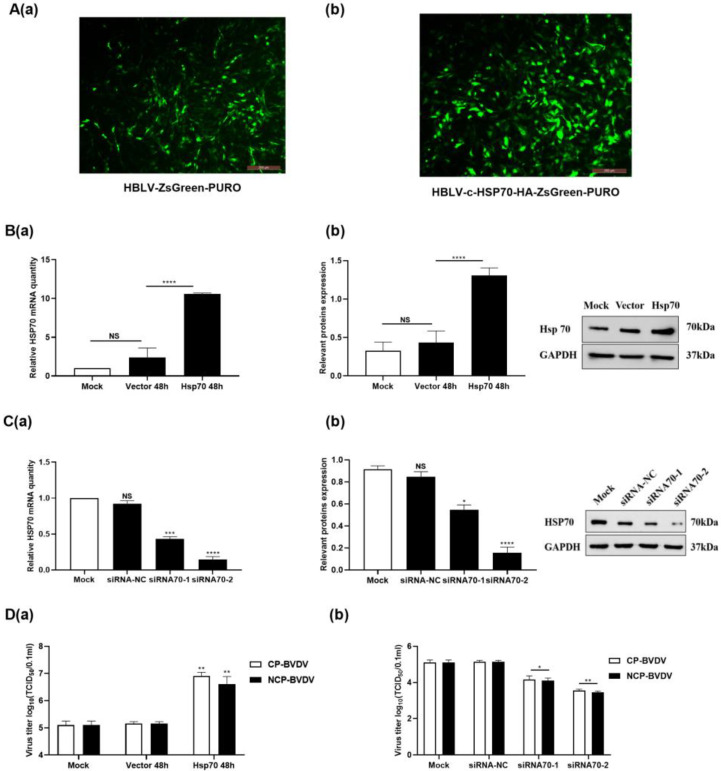
Hsp70 positively regulates the production of BVDV. (**Aa**): Fluorescence image of MDBK with lentiviral control. (**Ab**): Fluorescence image of MDBK cells with lentiviral Hsp70 infection. (**Ba**,**b**): Hsp70 mRNA and protein level in cells with lentiviral Hsp70 or control. (**Ca**,**b**): mRNA and protein expression of siRNA interference with Hsp70. (**Da**,**b**): Effect of lentivirus overexpressing Hsp70 and Hsp70 siRNA on BVDV viral titers. **** *p* < 0.0001, *** *p* < 0.001, ** *p* < 0.01, * *p* < 0.05, *n* = 3. Data are presented as mean ± SD.

To investigate the effect of Hsp70 on BVDV replication, lentiviruses overexpressing Hsp70 and Hsp70 siRNA were constructed. Fluorescence was observed in 80–90% of cells after being infected with the Hsp70 gene overexpressing lentivirus, and mRNA and protein levels of Hsp70 were also significantly higher than those of the control group (infected with empty lentivirus) (*p* < 0.05) (Figure 2A,B). These results indicated that Hsp70 was overexpressed in the majority of cells after Hsp70 lentivirus infection. After the transfecting of MDBK cells with siHsp70-1 and siHsp70-2, the mRNA and protein levels of the Hsp70 gene were significantly reduced compared to those in the control group (*p* < 0.05), indicating that both siHsp70-1 and siHsp70-2 could successfully interfere with the expression of Hsp70 (*p* < 0.05) (Figure 2C). MDBK cells were transfected with lentivirus overexpressing Hsp70 for 48 h and were then infected with (cp/ncp) BVDV. The virus titer in the culture supernatant was measured 48 h after infection. The results showed that the viral titer of the overexpression group was significantly higher than that of the control group. Similarly, cells were transfected with Hsp70 siRNA, and the virus titer in the culture supernatant was measured 48 h after virus infection. The result showed that the virus titer in the Hsp70 siRNA group was significantly lower than that in the control group (Figure 2D).

**Figure 3:**
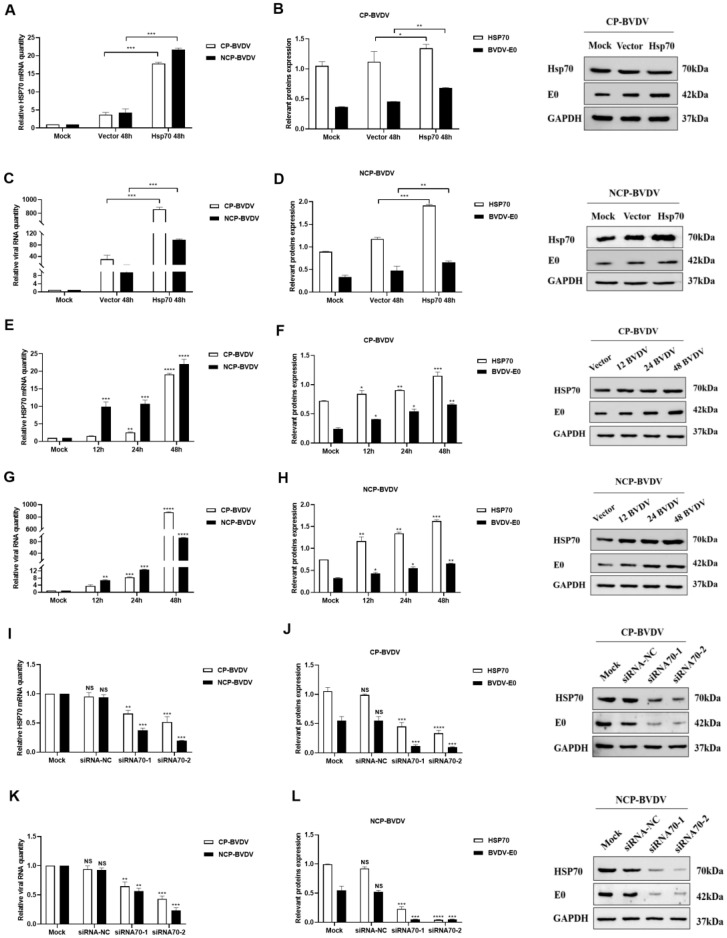
Effects of overexpression/RNA interference of Hsp70 on BVDV replication. (**A**–**D**): Expression of Hsp70 and viral mRNA and protein in Hsp70-overexpressed MDBK cells infected with cp/ncp viruses. (**E**–**H**): The expression of Hsp70 and viral mRNA and protein in MDBK at different time points after overexpression of Hsp70 and being infected with cp/ncp virus. (**I**–**L**): The expression of Hsp70 and virus mRNA and protein in MDBK after siRNA interference and being infected with cp/ncp viruses. **** *p* < 0.0001, *** *p <* 0.001, ** *p <* 0.01, * *p <* 0.05, *n* = 3. Data are presented as mean ± SD.

After infection with (cp/ncp) BVDV, mRNA and protein levels of Hsp70 and virus in the Hsp70 overexpression group were higher than those in the control group (*p* < 0.05). The expression levels of Hsp70 and virus in the ncp group were higher than those in the cp group (*p* < 0.05) (Figure 3A–H). After (cp/ncp) BVDV infection, the mRNA and protein levels of Hsp70 and virus in the siHsp70 group were significantly lower than those in the siRNA empty vector control group (*p* < 0.05). The effect of siHsp70-2 was more significant than that of siHsp-1 (*p* < 0.05) (Figure 3I–L). These results indicated that the overexpression of Hsp70 promoted the replication of (cp/ncp) BVDV, and the reduction in Hsp70 expression could inhibit (cp/ncp) BVDV replication.

### 3.3. Quercetin and Hsp70 Reduce BVDV-Induced Oxidative Stress

**Figure 4:**
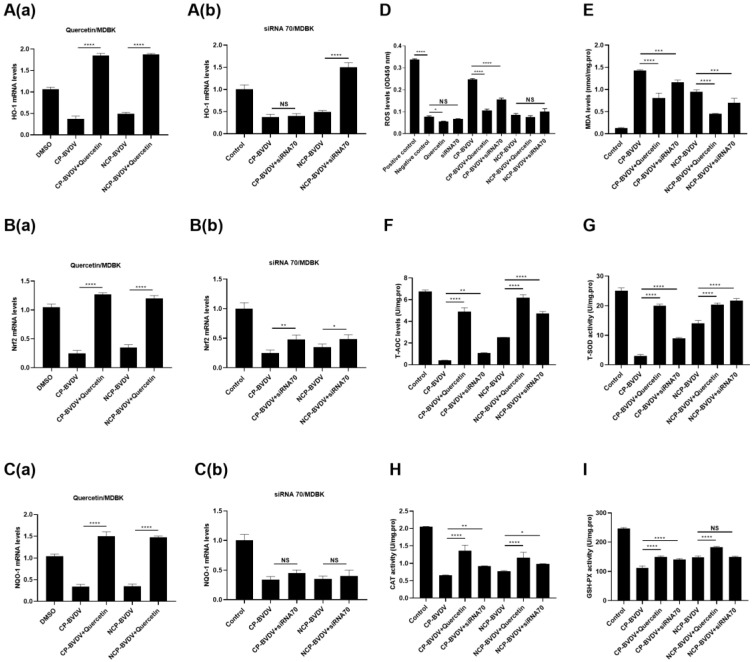
Effects of quercetin and Hsp70 siRNA on oxidative stress and expression of antioxidant genes in MDBK cells. (**Aa**–**Ca**): Effect of quercetin on the expression levels of HO-1, Nrf2, and NQO-1 in MDBK cells infected with (cp/ncp) BVDV. (**Ab**–**Cb**): Effects of siRNA Hsp70 on the expression levels of HO-1, Nrf2, and NQO-1 in MDBK cells infected with (cp/ncp) BVDV. *** *p <* 0.001, ** *p <* 0.01, * *p <* 0.05, *n* = 3. Data are presented as mean ± SD. (**D**): ROS level. (**E**): MDA level. (**F**): T-AOC level. (**G**): T-SOD activity. (**H**): CAT activity. (**I**): GSH-PX activity. **** *p* < 0.0001, *** *p <* 0.001, ** *p <* 0.01, * *p <* 0.05, *n* = 3. Data are presented as mean ± SD.

To investigate the effects of quercetin and Hsp70 on BVDV-induced oxidative stress, MDBK cells were infected with (cp/ncp) BVDV and then treated with quercetin or Hsp70 siRNA. Oxidative stress factors were measured subsequently. The results showed that quercetin could significantly increase the level of antioxidant genes compared with the control group (Figure 4(Aa–Ca)). By contrast, Hsp70 siRNA could significantly inhibit antioxidant gene expression (Figure 4(Ab–Cb)). ROS analysis showed that cp-BVDV could significantly stimulate ROS production (*p* < 0.05), while ncp-BVDV could not. When MDBK cells were pretreated with quercetin followed by (cp/ncp) BVDV infection, the production of ROS induced by cp-BVDV was significantly reduced (*p* < 0.05). Hsp70 siRNA had an inhibitory effect on cp-BVDV-induced ROS (*p* < 0.05), but not on ncp-BVDV-induced ROS. These results indicated that cp-BVDV could promote the production of ROS in MDBK cells, and when treated with quercetin or Hsp70 siRNA, ROS could be significantly reduced (Figure 4D). After infection with BVDV, MDA was increased while antioxidant enzymes were decreased (*p* < 0.05). When cells were treated with quercetin or Hsp70 siRNA, MDA production was significantly reduced (*p* < 0.05) (Figure 4E–I).

### 3.4. Quercetin and Hsp70 Affects ERK/P–ERK Pathway

**Figure 5:**
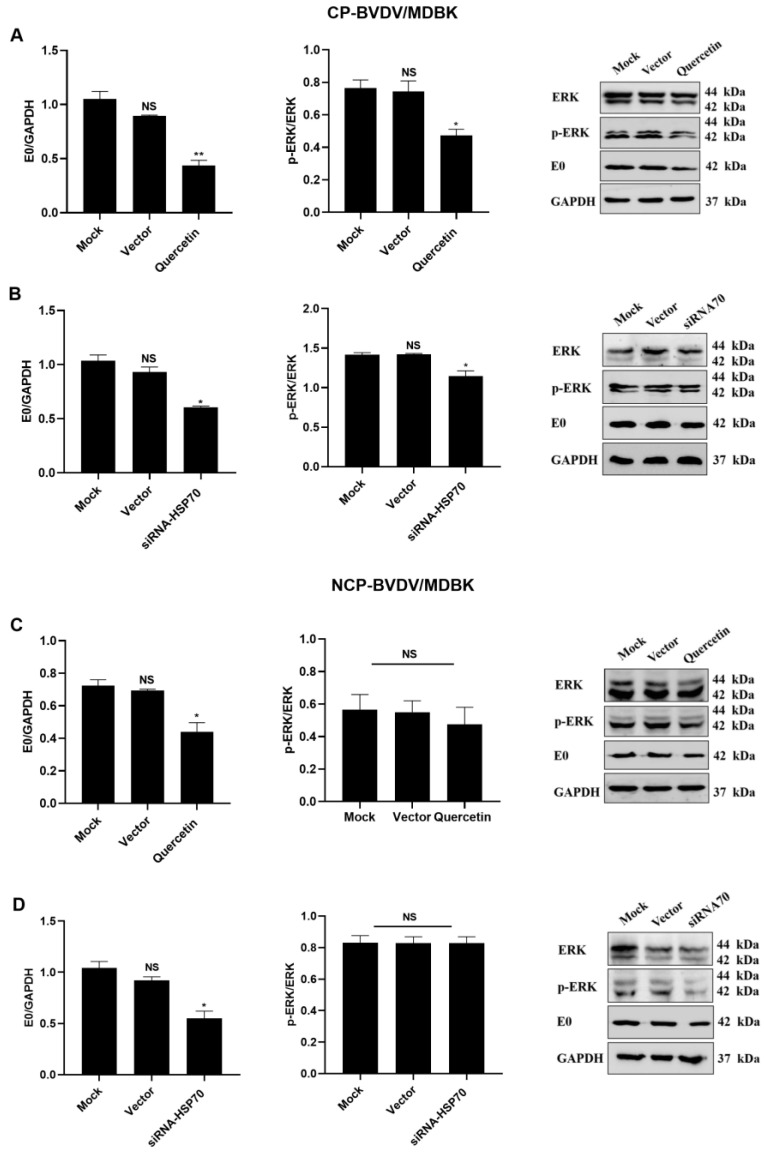
Quercetin and Hsp70 affect the ERK/P–ERK pathway. (**A**): The effect of cp-BVDV infection on the ERK/P–ERK pathway after adding quercetin. (**B**): The effect of cp-BVDV infection on the ERK/P–ERK pathway after transfection with Hsp70 siRNA. (**C**): Effects of ncp-BVDV on the ERK/P–ERK pathway adding quercetin. (**D**): The effect of ncp-BVDV infection on the ERK/P–ERK pathway after transfection with Hsp70 siRNA. ** *p* < 0.01, * *p* < 0.05, *n* = 3. NS: The difference is not significant. Data are presented as mean ± SD.

MDBK cells were treated with quercetin or siRNA and were then infected with (cp/ncp) BVDV. Subsequently, the phosphorylation level of ERK was examined. Treatment with quercetin or Hsp70 siRNA significantly reduced the phosphorylation level of ERK in the cp-BVDV-infected group (*p* < 0.05). In contrast, it did not significantly affect the phosphorylation level of ERK in the ncp-BVDV-infected group (*p* ˃ 0.05) (Figure 5). These results demonstrated that quercetin and Hsp70 could affect the phosphorylation level of ERK in cells infected with cp-BVDV.

### 3.5. Quercetin Inhibits Virus Replication in Mice

**Figure 6:**
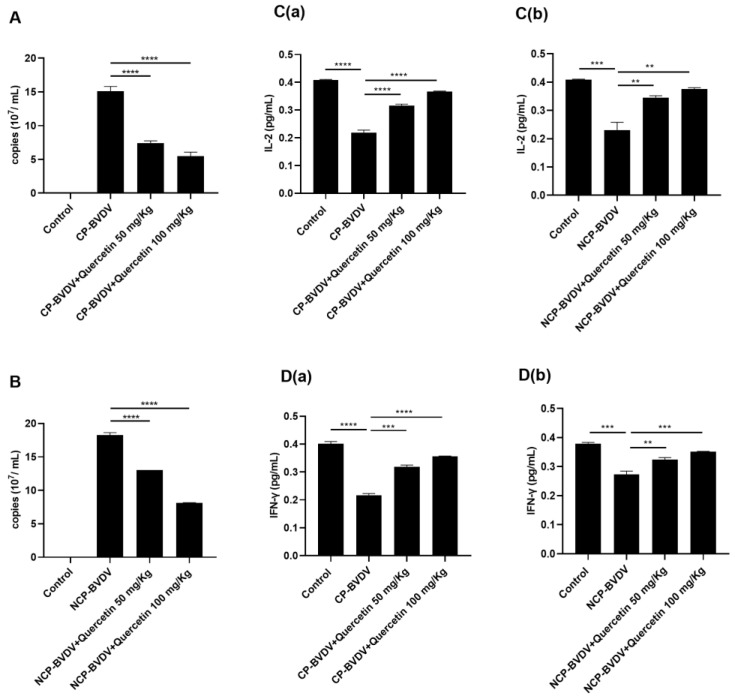
Quercetin affects viral replication and the expression levels of IFN-γ and IL-2 in BVDV-infected mice. (**A**): Quercetin against the cp-BVDV virus. (**B**): Quercetin against the ncp-BVDV virus. (**Ca**): Effect of quercetin on the level of IL-2 in serum of CP-BVDV-infected mice. (**Cb**): Effect of quercetin on the level of IL-2 in serum of NCP-BVDV-infected mice. (**Da**): Effect of quercetin on the level of IFN-γ in serum of CP-BVDV-infected mice. (**Db**): Effect of quercetin on the level of IFN-γ in serum of NCP-BVDV-infected mice. **** *p* < 0.0001, *** *p <* 0.001, ** *p <* 0.01, *n* = 3. NS: The difference is not significant. Data are presented as mean ± SD.

To determine the viral replication in vivo, we used qRT-PCR to measure the copy number of viral genes in the blood in a mouse infection model. On day 7 after BVDV infection, viral genes were detected in all cp BVDV-infected and ncp BVDV-infected mouse samples. Among them, the viral copy number in the ncp BVDV-infected group was higher than that in the cp BVDV-infected group. When treated with quercetin (50 mg/kg and 100 mg/kg), the viral copy numbers of ncp BVDV and cp BVDV were significantly decreased, indicating that quercetin inhibited the virus replication in BVDV-infected mice (Figure 6A,B).

### 3.6. Quercetin Alleviates the Reduction of IFN-γ and IL-2 in the Serum of BVDV-Infected Mice

To further investigate the effect of quercetin on cytokines in BVDV-infected mice, we measured IL-2 and IFN-γ in mouse serum by ELISA. Both IL-2 and IFN-γ were significantly decreased after infection with ncp BVDV and cp BVDV viruses. Treatment with quercetin (50 mg/kg and 100 mg/kg) significantly increased the production of IL-2 and IFN-γ in the serum of cp BVDV and ncp BVDV-infected mice. These results indicated that quercetin could relieve the immunosuppression caused by BVDV infection (Figure 6C,D).

### 3.7. Quercetin Inhibits Oxidative Stress and Increases the Expression of Antioxidant Enzymes In Vivo

**Figure 7:**
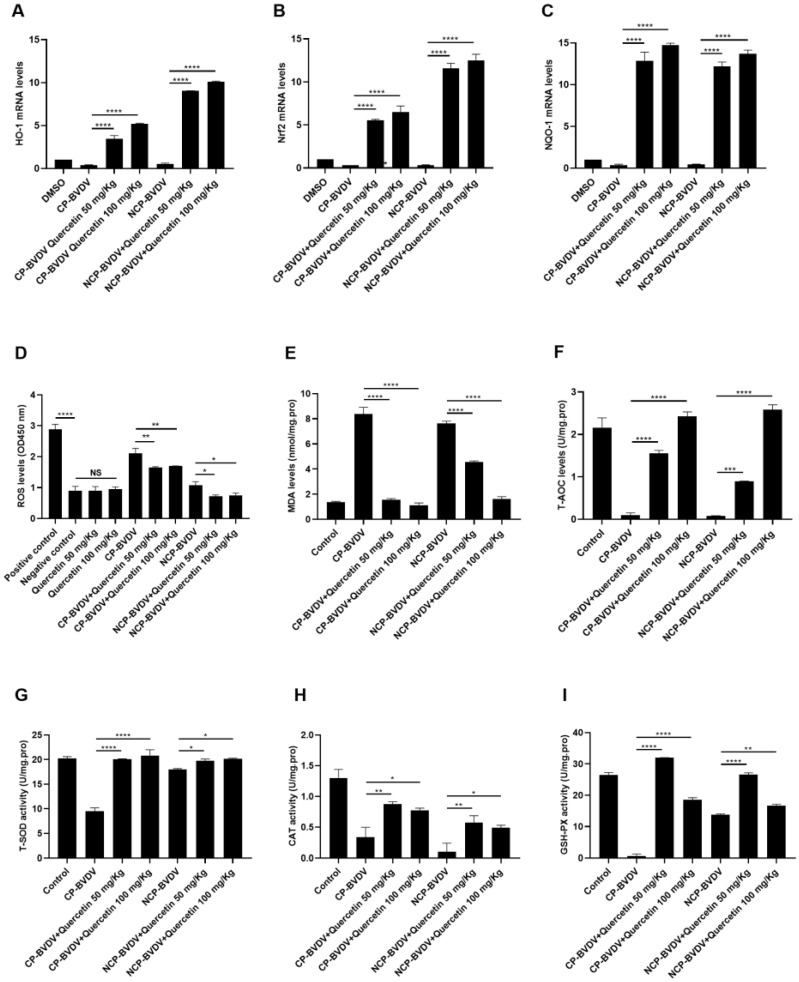
Effects of quercetin on oxidative stress and the expression of antioxidant genes in vivo. (**A**–**C**): Effect of quercetin on the expression levels of HO-1, Nrf2, and NQO-1 in MDBK cells infected with (cp/ncp) BVDV. (**D**): ROS level. (**E**): MDA level. (**F**): T-AOC level. (**G**): T-SOD activity. (**H**): CAT activity. (**I**): GSH-PX activity. **** *p* < 0.0001, *** *p* < 0.001, ** *p* < 0.01, * *p* < 0.05, *n* = 3. Data are presented as mean ± SD.

Quercetin (50 mg/kg and 100 mg/kg) up-regulated the expression of nuclear factor-related factor 2 (Nrf2), heme oxygenase-1 (HO-1), and oxidoreductase (NQO-1). Interestingly, the effect of quercetin on HO-1 and Nrf2 in the ncp BVDV-infected group was higher than that in the cp BVDV-infected group (Figure 7A–C). Oxidative stress (ROS) and malondialdehyde (MDA) production were significantly increased after infection with cp BVDV, while infection with ncp BVDV did not increase ROS, but caused an increase in MDA. When treated with quercetin (50 mg/kg and 100 mg/kg), oxidative stress (ROS) and MDA production were significantly reduced. In addition, ncp BVDV and cp BVDV infection inhibited the activity of antioxidant enzymes. When treated with quercetin (50 mg/kg), the activity of antioxidant enzymes could be significantly increased. Interestingly, when GXH-Px (NCP BVDV infection) and CAT were treated with 100 mg/kg of quercetin, the effect was not as prominent as that of 50 mg/kg (Figure 7D–I).

### 3.8. Quercetin Ameliorated BVDV-Induced Histopathological Changes

**Figure 8:**
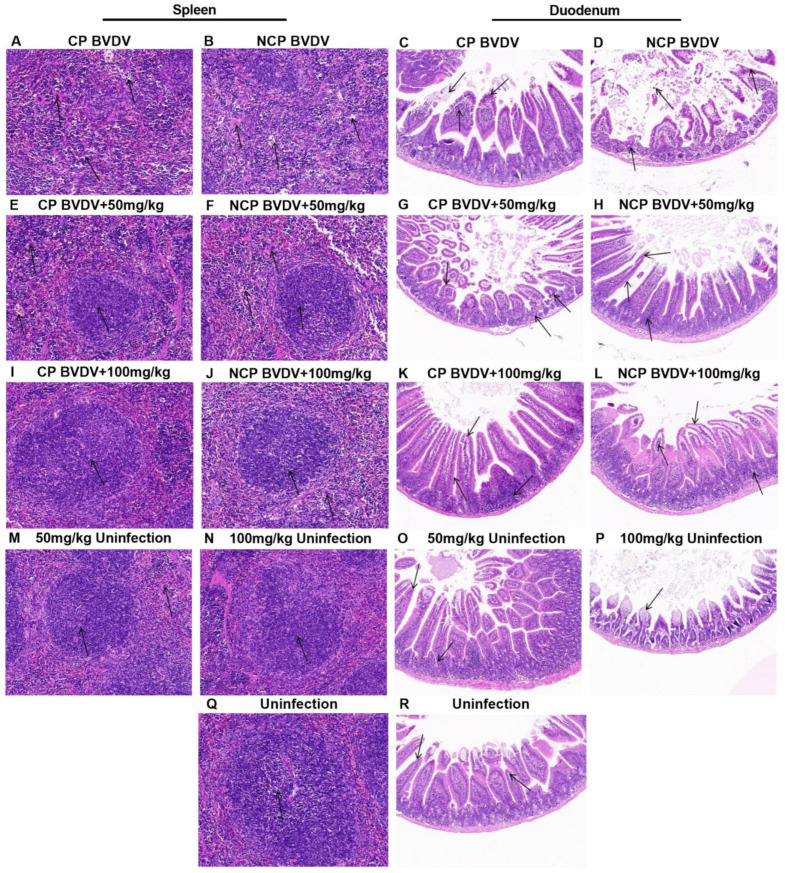
Histopathology of the spleen and duodenum at day 7 of post-infection. Images of the spleen [(**A**) CP BVDV; (**B**) NCP BVDV; (**E**) CP + 50 mg/kg; (**F**) NCP + 50 mg/kg; (**I**) CP + 100 mg/kg; (**J**) NCP + 100 mg/kg; (**M**) 50 mg/kg; (**N**) 100 mg/kg], duodenum [(**C**) CP BVDV; (**D**) NCP BVDV; (**G**) CP + 50 mg/kg; (**H**) NCP + 50 mg/kg; (**K**) CP + 100 mg/kg; (**L**) NCP + 100 mg/kg; (**O**) 50 mg/kg; (**P**) 100 mg/kg], negative control ((**Q**): spleen; (**R**): duodenum) (Hematoxylin and eosin, original magnification, 200×).

Histopathological examinations were performed on the spleen and duodenum of the infected mice on day 7 post-infection. Lymphocyte degeneration and necrosis, interstitial looseness and edema, and multinucleate giant cell infiltration were observed in the spleens of mice infected with cp (Figure 8A) and ncp (Figure 8B) BVDV. The duodenums of cp BVDV-infected mice showed the degeneration of mucosal epithelial cells, with intestinal tissue villi significantly disrupted, and inflammatory cell infiltration (Figure 8C). In contrast, the duodenums of ncp BVDV-infected mice showed necrosis and shedding of mucosal epithelial cells with inflammatory cell infiltration (Figure 8D). Compared with the virus-infected group, histopathological damage was alleviated after quercetin treatment (Figure 8E–L). In addition, no significant histological lesions were observed in quercetin-alone (50 mg/kg and 100 mg/kg) groups and the mock-infected mice groups (Figure 8M–R).

## 4. Discussion

Quercetin, a flavonoid widely distributed in the plant kingdom, causes multiple health-promoting effects in humans and animals that can be made into various additives with potential antiviral activities [7,8]. Studies have shown that Quercetin can bind viral non-structural protein 3 (NS3) and inhibit dengue virus infection by molecular docking prediction [27,28]. In addition, Quercetin inhibits Zika (ZIKV) virus replication in a dose-dependent manner in cell cultures and in mice [29]. Quercetin can also reduce the RNA copy number of Japanese encephalitis virus (JEV) and prevent virus replication in vitro [30]. These results demonstrate that quercetin has certain antiviral effects on members of Flaviviridae and is expected to be a potential anti-flavivirus compound. However, the underlying mechanisms of its antiviral effect remain unclear. Hsp70 is a promising therapeutic target for the development of novel small-molecule drugs. For example, Hsp70 can mediate ZiKV entry into host cells, replication, and egress. Yellow fever, West Nile virus, and Japanese encephalitis virus infections could be blocked when HSP70 inhibitors are used. Many studies have shown that quercetin can inhibit the expression of Hsp70 [15]. In addition, the antiviral activity of some drugs has been shown to be associated with the induction of specific Hsp70s [31]. However, the effect of quercetin on Hsp70 and BVDV in MDBK cells is still unclear. In order to determine the antiviral effect of quercetin on BVDV, we added quercetin before and after infecting MDBK cells with the BVDV virus. At the same time, its effect on Hsp70 expression was determined. Our results showed that quercetin inhibited the BVDV virus replication and the expression of Hsp70. Importantly, the effect of quercetin on BVDV replication may occur in the early stage of virus infection. Although studies have demonstrated that heat shock proteins are key cytokines for viral replication, the relationship between Hsp70 and BVDV has not yet been reported [32]. Therefore, we constructed lentivirus overexpressing Hsp70 and Hsp70 siRNA to determine further the effect of Hsp70 on BVDV replication. We infected cells with (cp/ncp) BVDV and found that overexpression of Hsp70 can increase virus replication. It is important to note that the virus expression level of cp-BVDV was significantly higher than that of ncp-BVDV at 48 h (*p* < 0.05). This could be due to the reasons that replication of cp-BVDV is more efficient than that of ncp-BVDV [33]. Then, we treated MDBK cells with Hsp70 siRNA to verify the effect of Hsp70 knockdown on BVDV replication and found that Hsp70 siRNA inhibited BVDV replication. These results indicated that high expression of Hsp70 can promote the replication of the BVDV virus, while inhibition of Hsp70 could inhibit the replication of the virus. Hsp70 expression could positively regulate the production of BVDV, and quercetin could inhibit the expression of Hsp70 in host cells.

Various studies have reported that the mechanistic pathway (ERK1/2, ROS, Nrf2, PI3K/AKT, MAPK) of action of quercetin is through its antioxidant potential [11]. Proteomic analysis shows that the production of large amounts of viral proteins during infection leads to cellular stress and elevated Hsp70 expression [26]. CP biotype can induce apoptosis in bovine embryonic turbinate cells in vitro, and in the early stage of the apoptosis process, intracellular ROS levels are elevated, suggesting the presence of oxidative stress [21]. As mentioned above, quercetin has strong anti-inflammatory and antioxidant effects. Therefore, we hypothesize that quercetin induces Hsp70 to affect BVDV replication through its antioxidant activities [24]. To test this hypothesis, we examined the effects of quercetin and Hsp70 siRNA on BVDV-induced oxidative stress, respectively. Our results showed that quercetin could reduce the oxidative stress caused by cp BVDV and promote the production of antioxidant enzymes. Knockdown of Hsp70 alleviated the oxidative stress caused by cp BVDV and inhibited the expression of antioxidant genes and the production of antioxidant enzymes. Interestingly, the expression of HO-1 in the ncp group was significantly higher than in the control group. Different viral infections have different regulatory effects on the expression of HO-1 in host cells [34]. Upregulation of HO-1 in ncp-BVDV-infected MDBK cells may be due to the activation of the antioxidant response signaling pathway. Alternatively, it may not be inhibited by miRNA, or it is affected by other regulatory mechanisms. These results indicated that quercetin and Hsp70 reduced BVDV-induced oxidative stress. So, the antiviral activity of quercetin may be associated with the induction of Hsp70.

It is well known that extracellular signal-regulated kinase (ERK) 1/2 plays a key role in cell signal transduction. ERK can promote the phosphorylation of cellular substrates to regulate cell growth, differentiation, death, and stress response and can regulate IL-2 production [11,35,36]. Importantly, the molecular target of quercetin is ERK [11]. Lim and other studies have shown that ERK is a kinase directly involved in the phosphorylation of Hsp70, and there is a positive correlation between the phosphorylation of Hsp70 and the activity of ERK [23]. ERK-dependent phosphorylation promotes the folding activity of Hsp70 and cell proliferation [37]. Conversely, the downregulation of Hsp70 promotes cell apoptosis [26]. Our previous studies have demonstrated that PD-1 blockade increases the level of p-ERK in BVDV-infected mice [1]. Therefore, we hypothesized that BVDV-induced ERK phosphorylation in MDBK cells could be closely related to quercetin or Hsp70. After interfering Hsp70 with quercetin or siRNA, we found that the level of p-ERK in the cp-BVDV infection group was significantly lower than that in the control group (*P* < 0.05), while the level in the ncp-BVDV infection group was not significantly different from the control group (*p ˃* 0.05). These results indicated that both quercetin and Hsp70 could affect the level of p-ERK induced by cp BVDV. Natural bioactive compounds are well known for their biological immunomodulatory activities. Quercetin is the first compound that specifically inhibits EBOV VP24 IFN-1-inhibitory function, restoring the IFN signaling cascade and leading to the blocking of viral infection [12]. Recent studies have reported that the immuno-stimulatory beneficial impact in the presence of quercetin is mediated by inducing Th-1-derived cytokine, IFN-γ, and inhibiting Th-2-derived cytokine, IL-4, and LPS/TNFR-induced TNF-α [11]. In addition, inhibition of Hsp70 can also enhance the induction function of IFN [16]. To further validate the effect of quercetin on BVDV replication and immunosuppression, we performed in vivo studies. Using our previously established mouse infection model, we determined the viral replication with IFN-γ and IL-2 levels in vivo. We showed that quercetin significantly inhibited viral replication in BVDV-infected mice. Meanwhile, quercetin alleviated the reduction of IFN-γ and IL-2 in serum in the BVDV-infected mice, which may be associated with inhibiting Hsp70 by quercetin.

In conclusion, we demonstrated that Hsp70 plays an important role in the life cycle of BVDV, especially in the RNA replication stage. Quercetin inhibits Hsp70, blocks BVDV infection in the early stage of virus infection and may also inhibit BVDV replication by inhibiting oxidative stress or ERK phosphorylation. In addition, quercetin alleviated the reduction of IFN-γ and IL-2 in the BVDV-infected mice. Quercetin ameliorated BVDV-induced histopathological changes. Therefore, quercetin is a promising anti-BVDV drug, and Hsp70 is an important target in the process of BVDV virus infection, both of which are beneficial for anti-BVDV virus therapy. These results provide the foundations for further investigations of the mechanism of immunosuppression by quercetin and Hsp70 during BVDV infection and new strategies for preventing and treating BVDV infections.

## Figures and Tables

**Table 1:** Primer amplification sequence of antioxidant gene.

Target Gene	Primer Sequence (5′–3′)	Product Length (bp)
Nrf2	F: ACCCAGTCCAACCTTTGTCGT	143
	R: GCGGCTTGAATGTTTGTCTTT	
NQO1	F: CGGCTCCATGTACTCTCTGC	183
	R: TCCAGGCGTTTCTTCCATCC	
HO-1	F: CAAGCGCTATGTTCAGCGAC	198
	R: TTGGTGGCACTGGCGATATT	
β-actin	F: CACCGCAAATGCTTCTAGGC	186
	R: TGTCACCTTCACCGTTCCAG	

## Data Availability

The raw data supporting the conclusions of this article will be made available by the authors, without undue reservation.
